# Splenic Injury Following Colonoscopy: A Case Report

**DOI:** 10.5811/cpcem.2021.2.50676

**Published:** 2021-05-25

**Authors:** Jason Wang, Heesun Choi, John Ashurst

**Affiliations:** * Touro University Nevada College of Osteopathic Medicine, Henderson, Nevada; † Kingman Regional Medical Center, Department of Emergency Medicine, Kingman, Arizona

**Keywords:** Colonoscopy, splenic hematoma

## Abstract

**Introduction:**

Colonoscopy is a commonly performed outpatient procedure with a low risk of complications. The most common complications seen in the postoperative period include hemorrhage and perforation. Infrequently, splenic injury can occur.

**Case Report:**

A 72-year-old male presented with a one-day history of left upper quadrant pain following colonoscopy. During the procedure he had two polyps removed along the transverse colon near the splenic flexure. There were no complications during the procedure or in the immediate post-operative period. On presentation to the emergency department, abdominal tenderness was present in the left upper quadrant without rebound, rigidity, or guarding. Point-of-care ultrasound of the abdomen demonstrated mixed hypoechoic densities confined to the splenic capsule, and computed tomography of the abdomen and pelvis with intravenous contrast noted a grade II/III splenic laceration without active extravasation. The patient was admitted for serial abdominal examination and labs.

**Conclusion:**

Splenic injury following colonoscopy is a rare complication of colonoscopy. Emergency providers should be aware of this possible complication, and acute management should include basic trauma care and consultation for possible intervention, if warranted.

## INTRODUCTION

Colonoscopy has become a routine diagnostic and treatment option for many colonic diseases and has a relatively low complication rate. The overall complication rate has been reported to range from 0.5% for routine colonoscopy and up to 1.8% for colonoscopy with polypectomy.^1^,^2^ The most common of these complications include hemorrhage and perforation.^3^ Less frequently, bacteremia, ileus, mesenteric tears, pneumothorax, pneumoperitoneum, pneumoscrotum and colonic volvulus can occur.^3^ We present a case of a splenic injury following colonoscopy with polypectomy that was successfully treated conservatively.

## CASE REPORT

A 72-year-old male presented due to left upper quadrant abdominal pain that radiated to the flank and constipation one day following a colonoscopy. He reported that he had not had a bowel movement or passed gas since the procedure. The indication for colonoscopy was chronic constipation. Following instructions from his surgeon, the patient had stopped taking clopidogrel and aspirin, both taken for moderate coronary artery disease, several days prior to the colonoscopy. During the procedure, the patient had two polyps removed along the transverse colon near the splenic flexure. Intraoperatively and postoperatively, there were no complications noted

Upon arrival to the emergency department, his vital signs were as follows: heart rate 66 beats per minute; blood pressure 113/59 millimeters of mercury; respiratory rate 16 breaths per minute; and temperature 98.7 degrees Fahrenheit. Upon examination, the patient appeared in no acute distress due to pain, and his skin and conjunctiva were without pallor. Abdominal examination revealed hypoactive bowel sounds, and there was tenderness to palpation in the left upper quadrant. No rebound, rigidity or guarding were seen on physical exam. No ecchymosis was noted in the left upper quadrant or the left flank.

**Image 1:**
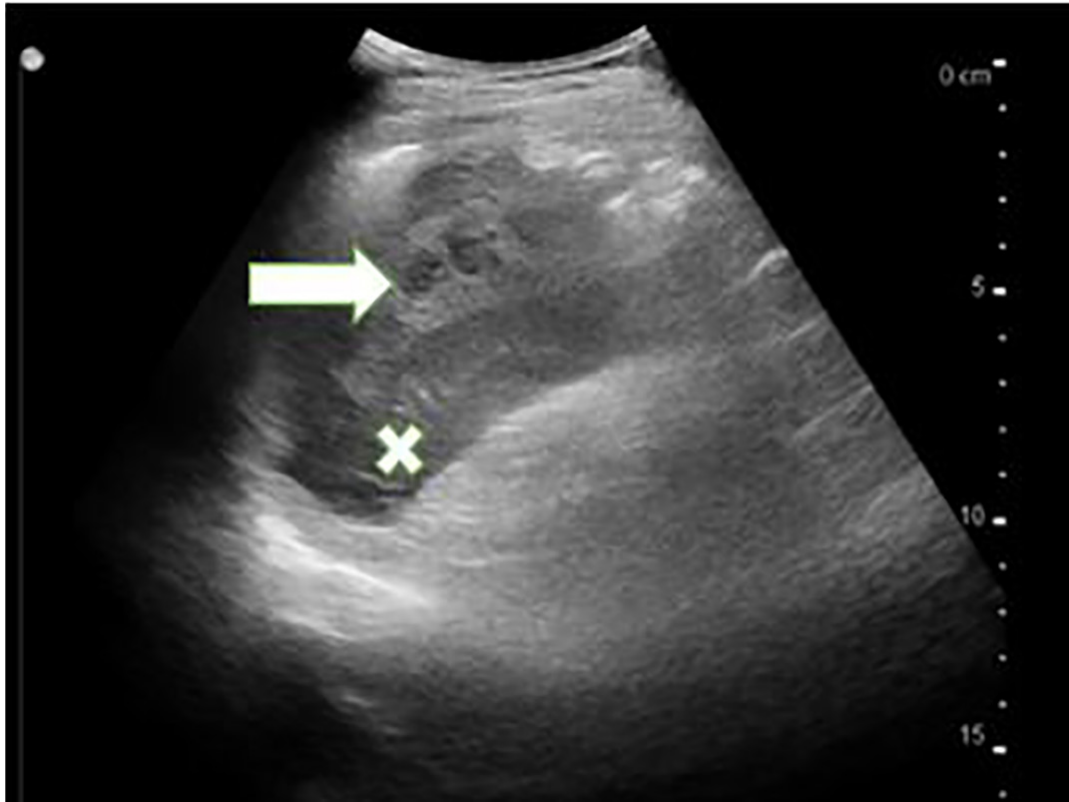
Point-of-care ultrasound of the spleen (X) with mixed hypoechoic densities within the splenic capsule (arrow).

**Image 2:**
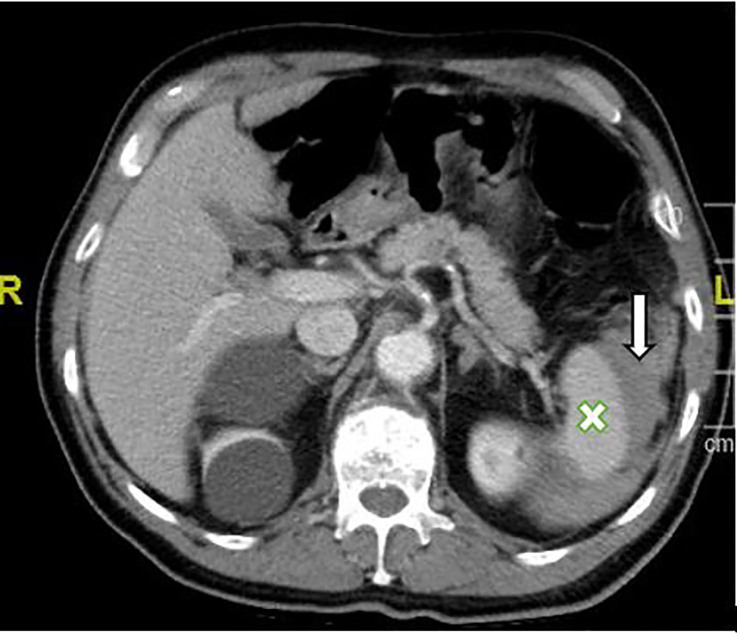
Computed tomography of the abdomen and pelvis with intravenous contrast depicting a perisplenic hematoma (arrow) with small amounts of blood products extending into the intraperitoneal space from the spleen (X).

A point-of-care ultrasound of the spleen demonstrated mixed hypoechoic densities within the splenic capsule (Image 1). Computed tomography (CT) of the abdomen and pelvis with intravenous contrast showed a perisplenic hematoma with small amounts of blood products extending into the intraperitoneal space (Image 2). There were also heterogeneously hyperdense blood products surrounding greater than 50% of the circumference of the spleen, consistent with a grade II/III splenic injury. Initial complete blood counts demonstrated hemoglobin 16.1 grams per deciliter (g/dL) (reference range 13.1 – 17.1 g/dL), hematocrit 47.3% (42–52%), and a white blood cell count 12,600 cells per microliter (cells/μL) (4,800 – 10,800 cells/μL). After consultation with general surgery, he was admitted for serial labs and abdominal examinations. He was discharged from the hospital three days later without requiring further intervention.

## DISCUSSION

Splenic injury following colonoscopy was first described in 1974 and is likely under-reported in the current literature.^4^–^6^ Common risk factors for splenic injury during colonoscopy include a technically demanding procedure, splenocolic adhesions, splenomegaly, underlying splenic disease, or polypectomy in the transverse colon as seen in this patient. Although the exact mechanism for injury is unknown, several theories have been developed.^7^ Excess traction upon the splenocolic ligament or adhesions can lead to partial capsular avulsion or splenic tears.^7^ Trauma following endoscopic navigation of the splenic flexure has also been described as a mechanism for injury.^7^

**CPC-EM Capsule**

What do we already know about this clinical entity?

*Although complications following colonoscopy occur at a rate of 0.5% to 1.8%, splenic injury is quite rare.*

What makes this presentation of disease reportable?

*This paper highlights the usage of point of care ultrasound as a diagnostic modality in those who present with abdominal pain following colonoscopy.*

What is the major learning point?

*Although rare, splenic injury following colonoscopy should be treated much like a splenic injury following trauma*

How might this improve emergency medicine practice?

*By keeping a broad differential diagnosis, the emergency medicine physician should employ early consultation and trauma intervention to improve patient outcomes.*

Typically, patients will present with abdominal pain or left shoulder pain (Kehr’s sign) on the same day as the procedure.^6^ However, a delayed presentation can occur and can lead to an increase in morbidity and mortality. In addition to physical exam, patients should be evaluated for signs of hypovolemia. While this patient initially presented with a blood pressure seemingly low for his age, this may have been caused by his daily use of lisinopril for chronic hypertension. Much as in blunt abdominal trauma, CT imaging should be considered the imaging modality of choice in patients who are hemodynamically stable. For those hemodynamically unstable, a focused abdominal sonography for trauma should be completed.

Treatment should follow the guidelines for the management of blunt traumatic splenic injuries. In the unstable patient with a splenic injury, exploratory laparotomy should be considered as the first-line treatment modality. In stable patients with active contrast extravasation from the spleen on CT, interventional radiology angioembolization should be considered after consultation. Those without active extravasation on the CT should be admitted for serial hemoglobin levels and abdominal examinations.

## CONCLUSION

An important part of obtaining a complete history includes eliciting any recent procedures as well as surgeries, including those performed in an outpatient setting. Although rare, splenic injury following outpatient colonoscopy is a complication that the emergency physician should include in the differential diagnosis for patients who present in the postoperative period. Early consultation, intervention, and appropriate trauma management should be used to avoid immediate and long-term morbidity and mortality.
